# Theoretical Investigation of HER and OER Electrocatalysts Based on the 2D R-graphyne Completely Composed of Anti-Aromatic Carbon Rings

**DOI:** 10.3390/molecules28093888

**Published:** 2023-05-05

**Authors:** Cuimei Li, Tianya Li, Guangtao Yu, Wei Chen

**Affiliations:** 1 Engineering Research Center of Industrial Biocatalysis, Fujian Provincial Key Laboratory of Advanced Materials Oriented Chemical Engineering, College of Chemistry and Materials Science, Fujian Normal University, Fuzhou 350007, China; lcm19@mails.jlu.edu.cn (C.L.); leechemistry@163.com (T.L.); 2 Academy of Carbon Neutrality of Fujian Normal University, Fuzhou 350007, China; 3 Laboratory of Theoretical and Computational Chemistry, Institute of Theoretical Chemistry, Jilin University, Changchun 130023, China

**Keywords:** 2D material R-graphyne, hydrogen evolution reaction (HER), oxygen evolution reaction (OER), electrocatalysts, DFT calculations

## Abstract

Based on the DFT calculations, two-dimensional (2D) R-graphyne has been demonstrated to have high stability and good conductivity, which can be conducive to the relevant electrocatalytic activity of the material. Different from the poor graphene, R-graphyne, which is completely composed of anti-aromatic structural units, can exhibit certain HER catalytic activity. In addition, doping the TM atoms in Group VIIIB can be considered an effective strategy to enhance the HER catalytic activity of R-graphyne. Particularly, Fe@R-graphyne, Os@R-graphyne, Rh@R-graphyne and Ir@R-graphyne can exhibit higher HER catalytic activities due to the formation of more active sites. Usually, the shorter the distance between the TM and C atoms is, the better the HER activity of the C-site is. Furthermore, doping Ni and Rh atoms of Group VIIIB can significantly improve the OER catalytic performance of R-graphyne. It can be found that ΔG_O*_ can be used as a good descriptor for the OER activities of TM@R-graphyne systems. Both Rh@R-graphyne and Ni@R-graphyne systems can exhibit bifunctional electrocatalytic activities for HER/OER. In addition, all the relevant catalytic mechanisms are analyzed in detail. This work not only provides nonprecious and highly efficient HER/OER electrocatalysts, but also provides new ideas for the design of carbon-based electrocatalysts.

## 1. Introduction

Electrocatalytic water splitting for hydrogen production has great prospects in solving the global energy crisis caused by energy depletion and environmental pollution [1,2,3]. Electrolytic water splitting includes two half reactions: hydrogen evolution reaction (HER) and oxygen evolution reaction (OER). Owing to sluggish kinetics, electrocatalysts are required to reduce the overpotentials of these two reactions and improve the reaction kinetics. In previous reports, some HER or OER electrocatalysts have been designed and developed [4,5,6,7,8,9]. So far, the benchmark electrocatalysts for HER and OER are Pt [10] and IrO_2_/RuO_2_ [11,12], respectively. However, the scarcity and high cost of these precious metals have greatly hindered their large-scale applications. Therefore, many efforts have been made to design highly efficient HER/OER electrocatalysts by minimizing the use of precious metals.

At present, two-dimensional (2D) materials have attracted great attention from researchers because their unique planar structure with atomic thickness can bring some obvious advantages to catalytic reactions, such as providing a larger specific surface area and endowing them with abundant exposed active sites, allowing them to easily combine with other materials and affording them good catalytic activity by introducing defects or heteroatoms. Until now, some 2D materials have been reported as promising candidates for electrocatalytic HER/OER reactions, such as transition metal complexes ((e.g., TMS_2_ (TM = Fe, V and Mo) [13,14,15], TMC_2_ (TM = Ti, V, Nb, Ta and Mo) [16] and TMN_2_ (TM = Ir and Rh)) [17], layer metal triphosphide-based materials (e.g., GeP_3_, SnP_3_ and FeP_3_) [18,19,20], layered double hydroxides (LDHs) [21,22], graphene-like systems (e.g., BSi_n_ (n = 1–4), GeSi/SnSi/SnGe, g-GeC and GaN) [23,24,25,26], MBenes (e.g., Ti_2_B_2_O_2_, Mo_2_B_2_O_2_ and W_2_B_2_O_2_) [27], cobalt-ion-doped MXenes (e.g., Ti_2_CT_x_, Cr_2_CT_x_, and V_2_CT_x_) [28], metal-organic frameworks (MOFs) [29,30] and transition-metal-doped C_x_N_y_ nanomaterials (e.g., CN, C_2_N, C_9_N_4_, g-C_3_N_4_ and pc-C_3_N_2_) [31,32,33,34,35,36].

Moreover, 2D carbon-based materials are becoming a rising star in the field of electrocatalytic water splitting due to their low cost, abundance, easy functionalization and the tunability of their structure and components. Among them, graphene can be considered as one of the most important members in the 2D carbon-based materials family. As is well known, some graphene-based HER or OER electrocatalysts have been designed [37,38,39,40,41], although 2D pristine graphene exhibits electrochemical inertness. For example, graphene doped with non-metallic heteroatoms (including N, S, P, O and B) can show significantly increased HER catalytic activity [37]. The N-doped graphene-supported transition metal Co can also exhibit good electrocatalytic activity for HER [38]. In addition, it was found that hydroxyl-group-modified single metal atoms (Ni, Co or Fe) loaded on defective graphene can have high OER electrocatalytic activity [39]. Good OER catalytic performance can also be observed in the 2D N-doped graphene system embedded with the bimetallic FeCu [40]. Moreover, the 2D NiS/graphene heterostructure can even show high HER and OER activities in alkaline solution at the same time [41]. 

Different from the previous investigations, in this study we focus on another important member of the 2D carbon-based material family, namely R-graphyne [42,43], which is composed of four-membered rings and sixteen-membered rings. Similar to graphene, R-graphyne can also possess a π-conjugated structure, in which the relevant C atoms adopt sp^2^- or sp-hybridization. However, the difference is that the former is composed of aromatic six-membered carbon rings conforming to the (4n + 2) rule, while the latter is completely composed of anti-aromatic carbon rings conforming to the 4n rule. The existence of anti-aromaticity in all the structural units in R-graphyne could make the relevant carbon atoms show good reactivity, which should be significantly different from the case of graphene. Therefore, 2D R-graphene is highly expected to have potential applications in electrocatalytic reaction processes (such as HER). However, as far as we know, the application of R-graphyne in electrocatalytic water splitting has never been reported, in spite of its importance and significance. 

In this study, we intend to investigate the HER/OER catalytic performance of R-graphyne. As expected, this kind of unique 2D nanostructure composed entirely of anti-aromatic carbon rings can endow R-graphyne with certain HER catalytic activity, which is much better than the inert graphene. Furthermore, we propose an effective strategy through doping Group VIIIB elements to further enhance the catalytic activity of HER and OER. Among them, the doping of Fe, Os, Rh and Ir can more effectively improve the HER catalytic activity of R-graphyne, demonstrated by the appearance of more highly active sites. In contrast, embedding Ni and Rh atoms can induce considerably higher OER catalytic activity, where their calculated overpotentials can be even lower than that of the state-of-the-art IrO_2_ catalyst [12]. It is easy to find that Rh@R-graphyne and Ni@R-graphyne can exhibit bifunctional electrocatalytic activity for HER/OER. Obviously, this work can provide a new direction to realize high-performance and low-cost electrocatalysts for HER and OER by designing low-dimensional nanostructures completely composed of anti-aromatic rings.

## 2. Results and Discussion

### 2.1. Structure, Stability, Electronic Property and HER Electrocatalytic Activity of Pristine R-graphyne

**Figure 1:**
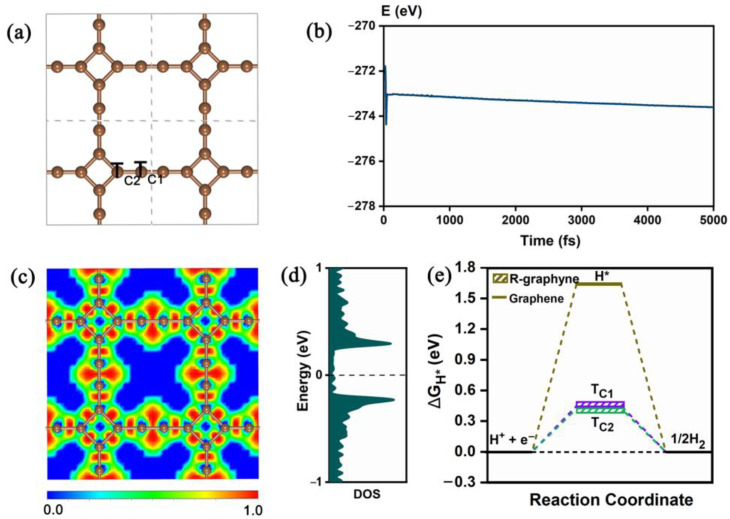
(**a**) The optimized structure of 2D R-graphyne and the obtained adsorption sites of H*, (**b**) the total energy curve of ab initio molecular dynamics (AIMD) simulation for R-graphyne at 500 K, where the inset is the configuration after 5000 fs, (**c**) the calculated electron location function (ELF) of the R-graphyne, (**d**) the density of states (DOS) of R-graphyne, where the Fermi level is set as 0, and (**e**) the calculated ΔG_H*_ values for the different adsorption sites of R-graphyne and graphene at equilibrium potential.

The dynamically and mechanically stable R-graphyne with a space group of P4/MMM can be considered to be composed of a four-membered carbon ring (C_4_) and a sixteen-membered carbon ring (C_16_), as shown in Figure 1a. The lattice parameters of the primitive unit cell are a = b = 6.011 Å. There are three nonequivalent C-C bonds, labeled as d_1_, d_2_ and d_3_ (Figure S1), with the bond lengths of 1.242, 1.351 and 1.463 Å, respectively, which is consistent with the previous work [42]. 

In this study, we performed an ab initio molecular dynamics (AIMD) simulation [44] at 500 K with a time constant of 5000 fs to evaluate the thermal stability of R-graphyne. It is clear that there is a small energy fluctuation, and the structural integrity of the original atomic configuration can be maintained after a time interval of 5 ps (Figure 1b), indicating the good thermal stability of R-graphyne. Furthermore, we calculated the electron localization function (ELF) [45] to illustrate the type of chemical bonding between two adjacent C atoms. As shown in Figure 1c, the red regions of the ELF diagram demonstrate the formation of strong covalent bonds and highlight the stability of R-graphyne. In addition, we investigated the electronic property of R-graphyne by calculating the density of states (DOS). As shown in Figure 1d, the metallic behavior can be observed in R-graphyne, where the correlative states cross the Fermi level, indicating good conductivity. It is well known that good conductivity can be beneficial to the relevant electrocatalytic activity of the material. 

Subsequently, we investigated the HER electrocatalytic performance of R-graphyne by calculating the free energy of hydrogen adsorption (ΔG_H*_). For comparison, the well-known 2D graphene was also considered. It has been well proven that the catalytic activity of the HER closely correlates with the adsorption energy of a single hydrogen atom on the material’s surface [46]. Therefore, ΔG_H*_ can be identified as a representative indicator to measure HER activity [13,18,23,30,47]. Usually, the smaller absolute value of ΔG_H*_ means a better HER catalytic activity of the material [46]. The calculation formula of ΔG_H*_ is provided in the section on computational methods. 

**Figure 2:**
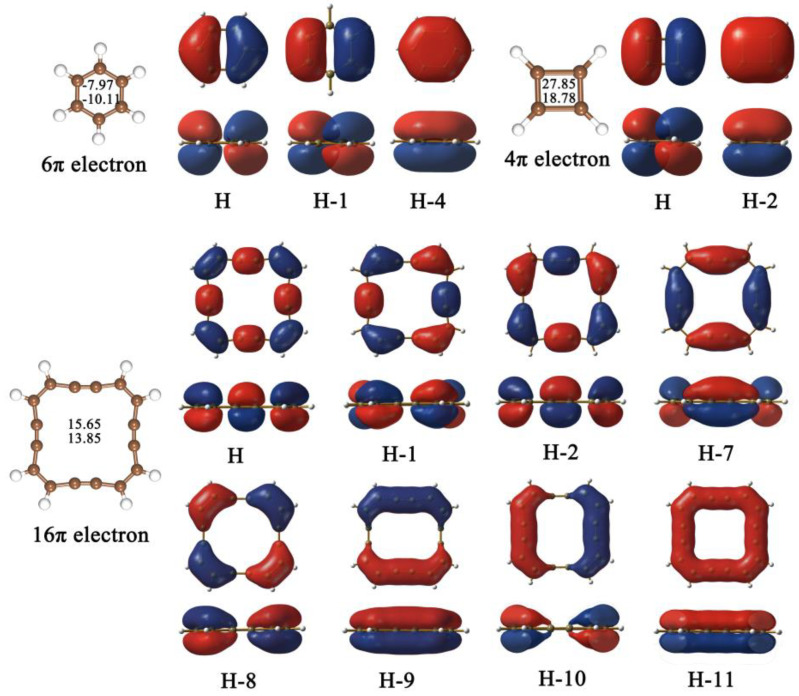
The calculated NICS(0)/NICS(1) values at the center of the six-, four- and sixteen-membered rings, as well as the corresponding π molecular orbitals (top and side views).

As shown in Figure 1e, two different adsorption sites (namely, T_C1_ and T_C2_) can be obtained on the 2D R-graphyne, and the calculated ΔG_H*_ values are 0.450 eV and 0.423 eV, respectively, indicating a certain catalytic activity of the HER. Obviously, compared with the inert HER activity of graphene (1.642 eV), the R-graphyne can show a good trend of change in HER catalytic performance, although both of them can be regarded as 2D π-conjugated structures composed of sp^2^- or sp-hybridized C atoms. This can be mainly due to the different structural units they are composed of. Specifically, graphene consists of aromatic six-membered carbon rings meeting the (4n + 2) rule of π electrons, which can be reflected well by the relevant molecular orbitals (Figure 2). Comparatively, R-graphyne is composed of four-membered and sixteen-membered carbon rings with anti-aromatic characteristics satisfying the 4n rule, which can also be reflected by the molecular orbitals of the related carbon rings (Figure 2). Furthermore, we have also carried out the NICS calculations, which can usually be used as a reliable indicator to determine the aromaticity [48]. Indeed, the aromaticity of six-membered carbon rings (−7.97/−10.11) and the anti-aromaticity of four-membered (27.85/18.78) or sixteen-membered (15.65/13.85) carbon rings can be further supported by the calculated negative and positive NICS(0)/NICS(1) values, respectively (Figure 2). Clearly, compared with the aromatic case of graphene, the presence of anti-aromaticity can bring more active C atoms, which leads to a certain HER catalytic performance of R-graphyne. Designing the 2D carbon allotrope completely composed of anti-aromatic structural units can be considered as an effective strategy to boost the HER catalytic activity of related carbon materials.

### 2.2. The HER Performance of TM@R-graphyne (TM = Fe, Ru, Os, Co, Rh, Ir, Ni, Pd and Pt)

From the above discussions, we found that the pristine R-graphyne can exhibit certain HER catalytic activity, which is mainly due to the anti-aromaticity of its structural unit rings. However, based on the fact that the calculated ΔG_H*_ values are not close to zero, we propose a promising approach through embedding the Group VIIIB elements (i.e., Fe, Co, Ni, Ru, Rh, Pd, Os, Ir and Pt) into R-graphyne with its unique structure to construct TM@R-graphyne to effectively improve the performance of the HER.

As shown in Figure S1, two possible doping sites of transition metal (TM) atoms are considered, that is, the TM atom is connected with four C atoms at the corner of the sixteen-membered ring (S1) and the TM is located over the top of the four-membered ring (S2). The calculated results show that configuration S1 can be much lower in energy than configuration S2, indicating the higher structural stability. Therefore, doping configuration S1 is considered in this study. It can be found that the planar configuration of R-graphyne can be well maintained after embedding the TM atoms of Group VIIIB (Figure S2). The calculated lattice parameters of TM@R-graphyne can be in the range of a = b = 11.934~11.994 Å (Table S1), which are comparable to the corresponding 2 × 2 supercell unit of the pristine R-graphyne. In addition, the calculated binding energies of TM-doped R-graphyne systems can be largely negative in the range of −2.509~−4.773 eV (Table S1), indicating that these VIIIB TM atoms can be stably anchored in R-graphyne. We have also performed the AIMD calculations for the TM@R-graphyne structure at 500 K for 5 ps (Figure S3). It can be found that the energy of TM@R-graphyne oscillates only in a small range during the AIMD simulation and the geometric structure can be basically maintained, indicating high thermal stability. Moreover, we investigate the electronic property of TM@R-graphyne by calculating the corresponding density of states (DOS). As shown in Figure S4, the metallic behavior can still be observed in these TM@R-graphyne systems, which suggests good conductivity. High stability and good conductivity can inspire us to explore the relevant electrocatalytic performance of TM@R-graphyne.

**Figure 3:**
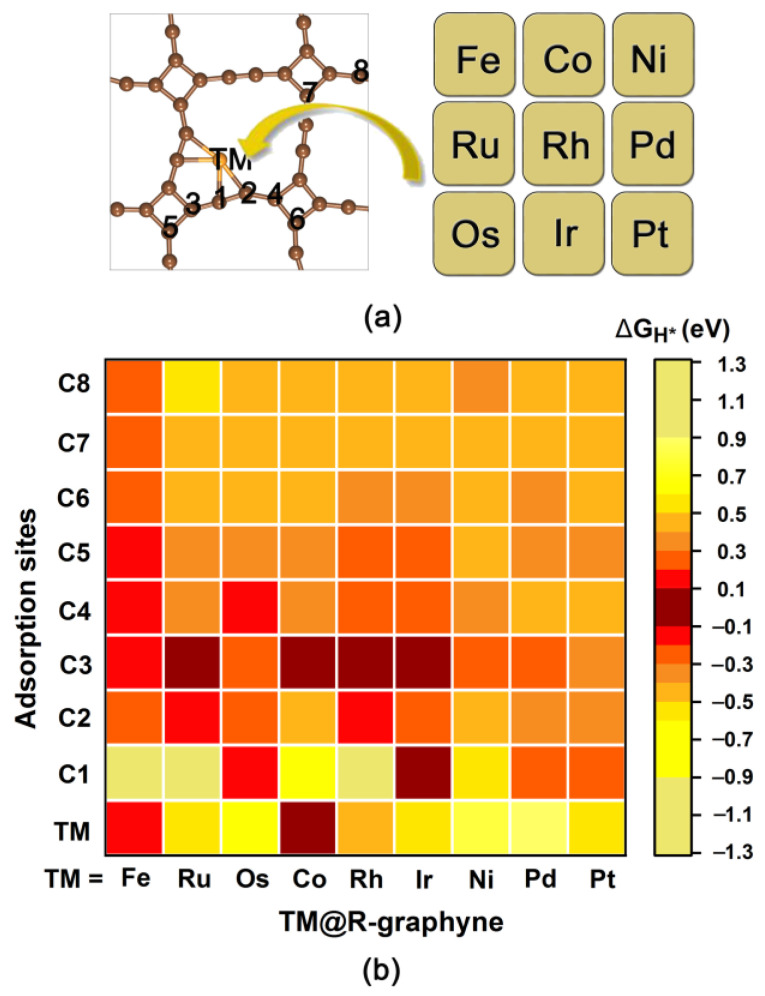
(**a**) The optimized structures of TM@R-graphyne (TM = Fe, Co, Ni, Ru, Rh, Pd, Os, Ir and Pt), where the obtained adsorption sites of H* are marked. (**b**) The calculated ΔG_H*_ values for different adsorption sites on TM@R-graphyne at equilibrium potential. Note that the HER catalytic performance can be indicated by the distinct colors, where the darker red color means higher activity.

Here, we investigate the HER catalytic activity for a series of TM@R-graphyne systems (TM = Fe, Ru, Os, Co, Rh, Ir, Ni, Pd and Pt). A total of nine possible adsorption sites over C atoms and TM atom are considered, namely T_C1~_T_C8_ and T_TM_, respectively (Figure 3a). Initially, we evaluate the HER catalytic performance of R-graphyne doped with Fe, Ru and Os atoms in the same column of Group VIIIB by calculating the ΔG_H*_ values of relevant possible adsorption sites (Figure 3b and Table S2). Our computed results reveal that when doping Fe atom into R-graphyne, almost all the C-sites (except for T_C1_) can exhibit much smaller ΔG_H*_ values in the range of −0.140~0.250 eV. Specifically, the calculated ΔG_H*_ values of T_C2,_ T_C3,_ T_C4_, T_C5_, T_C6_, T_C7_ and T_C8_ sites on Fe@R-graphyne can be as small as 0.239, −0.140, 0.122, 0.181, 0.235, 0.250 and 0.237 eV, respectively, uniformly suggesting considerably high HER catalytic activity. Moreover, the T_Fe_ site can also exhibit considerably high HER activity, in view of the small ΔG_H*_ value of 0.142 eV. Clearly, Fe@R-graphyne can possess excellent HER catalytic activity, much higher than the pristine R-graphyne, due to the appearance of more highly active sites with much smaller ΔG_H*_ values.

When doping Ru and Os atoms in the higher period, high HER activity can also be observed, although the number of highly active sites on Ru@R-graphyne and Os@R-graphyne decreases slightly compared with Fe@R-graphyne. Specifically, doping Ru can make the ΔG_H*_ values of some C-sites become smaller, including T_C2_ (−0.104), T_C3_ (−0.014), T_C4_ (0.316) and T_C5_ (0.328 eV), all of which can possess higher HER activity than the pristine R-graphyne. In particular, some C atoms around the Ru-doped site, including T_C2_ (−0.104 eV) and T_C3_ (−0.014 eV), can exhibit near-zero ΔG_H*_ values, suggesting their considerably high HER catalytic activities. In contrast, the absolute ΔG_H*_ values of the remaining C-sites (except for T_C1_) are in the range of 0.425~0.503 eV, all of which are comparable to the corresponding those of pristine R-graphyne, implying that these C-sites can maintain the original HER activity. Similarly, the doping of Os can also bring the smaller ΔG_H*_ values to most of the C-sites, including T_C1_ (−0.140 eV), T_C2_ (0.272 eV), T_C3_ (−0.263 eV), T_C4_ (0.196 eV) and T_C5_ (0.334 eV), suggesting their better HER activities than the corresponding ones on the pristine R-graphyne. Comparatively, the calculated ΔG_H*_ values of the remaining C-sites on Os@R-graphyne are in the range of 0.461~0.496 eV, indicating that these C-sites can maintain the original HER activity. It can be found that for Ru@R-graphyne and Os@R-graphyne, the closer distances between the relevant C atom and the TM-doping site can generally bring a better HER catalytic activity of the C-site, mainly because it can be activated more effectively. In addition, with the increase in the periodic number of the doped TM atom, the adsorption strength of H* at the metal site will increase, and the corresponding HER catalytic activity will gradually decrease, as revealed by the calculated ΔG_H*_ results of three TM sites including T_Fe_ (0.142 eV), T_Ru_ (−0.501 eV) and T_Os_ (−0.778 eV).

Overall, a series of TM@R-graphyne systems (TM = Fe, Ru and Os) can uniformly possess high HER catalytic activity, in view of the formation of highly active sites with much smaller ΔG_H*_ values. In particular, doping Fe can induce higher HER catalytic activity due to the presence of more active sites. 

Subsequently, we investigate the HER catalytic activity of the R-graphyne systems doped with Co, Rh and Ir atoms with more valence electrons in the same column of Group VIIIB by considering the relevant possible adsorption sites (Figure 3a,b). Our computed results show that for Co@R-graphyne, both the T_C3_ (0.068) and T_Co_ (0.069 eV) sites can have the near-zero ∆G_H*_ value, indicating the remarkable HER activity of these two sites. The absolute values of ΔG_H*_ for other C-sites (except for T_C1_) are in the range of 0.369~0.449 eV, indicating that their HER activity can be superior to or comparable to the corresponding activity of pure R-graphyne. As for Rh@R-graphyne, most of the C-sites (including T_C2,_ T_C3_, T_C4,_ T_C5_ and T_C6_) can also exhibit enhanced HER activity compared with the pristine R-graphyne, as reflected by their much smaller ΔG_H*_ values in the range of −0.118~0.399 eV. Particularly, some C-sites near the Rh-doping site can even present very small ∆G_H*_ values, namely T_C2_ (−0.118 eV) and T_C3_ (−0.037 eV), indicating the considerably high HER activity. In contrast, the absolute ΔG_H*_ values of all C-sites on Ir@R-graphyne can be in the range of 0.055~0.449 eV, which are smaller than or close to the corresponding ones on the pure R-graphyne. Among them, the calculated ΔG_H*_ values of carbon sites (including T_C1_, T_C2_, T_C3_ and T_C4_) near the Ir-doping site can be as small as −0.005, 0.246, −0.099 and 0.241 eV, respectively, reflecting considerably high HER activity. Similarly, with the increase inthe periodic number of TM atoms from Co to Ir, the HER catalytic activity of the TM site can present a gradually decreasing trend, which is reflected in the corresponding ΔG_H*_ results of three TM sites including T_Co_ (0.069 eV), T_Rh_ (−0.497 eV) and T_Ir_ (−0.581 eV). 

Obviously, the HER activity on 2D R-graphyne can also be effectively improved by doping Co, Rh and Ir atoms with more valence electrons, and especially embedding Ir can more effectively enhance the HER activity due to the presence of more highly active sites. Similarly, it can also be observed that in this series of systems, the shorter the distance between the TM and C atoms, the higher the HER activity of the T_C_ site.

Next, we continue to explore the HER catalytic performance of R-graphyne systems doped with Ni, Pd and Pt atoms in the same column, which can possess the most valence electrons in Group VIIIB. Our computed results show that due to the formation of active sites, doping Ni, Pd and Pt can improve the HER activity of R-graphyne, although their HER catalytic activity can be lower than that of R-graphyne doped with other TM atoms in Group VIIIB (TM = Fe, Ru, Os, Co, Rh and Ir). Specifically, the T_C3_ site (0.210 eV) on Ni@R-graphyne, the T_C1_ (0.240 eV) and T_C3_ (0.282 eV) sites on Pd@R-graphyne and the T_C1_ site (0.267 eV) on Pt@R-graphyne can exhibit relatively high HER activity. The calculated absolute ΔG_H*_ values of the remaining C-sites on TM@R-graphyne can be in the range of 0.372~0.600 eV, 0.351~0.421 eV and 0.303~0.429 eV for TM = Ni, Pd and Pt, respectively, most of which can be comparable to the corresponding values of pristine R-graphyne, suggesting that these C-sites can basically maintain the original HER activity. In addition, all three relevant TM sites (Ni, Pd and Pt) can have largely positive ΔG_H*_ values in the range of 0.586~0.887 eV (Table S2), indicating relatively poor HER activity. 

Furthermore, we also discussed the effect of increasing the valence electron number of TM atoms on the HER catalytic activity of the doped R-graphyne systems. Specifically, with the increase in the valence electron number of 3d TM atoms (Fe, Co and Ni), the HER activity of TM@R-graphyne will reduce owing to the decrease in highly active sites. In contrast, the 4d Ru- and Rh-doped R-graphyne systems can have similar HER activity, which are better than the parallel Pd-doped R-graphyne. Similarly, 5d Os- and Ir-doped R-graphyne systems can also exhibit comparable HER catalytic activity, which are higher than the parallel Pt@R-graphyne. Among them, Os@R-graphyne, Ir@R-graphyne, Rh@R-graphyne and especially Fe@R-graphyne can exhibit higher HER activity than the remaining TM@R-graphyne systems, due to the presence of more active sites. 

**Figure 4:**
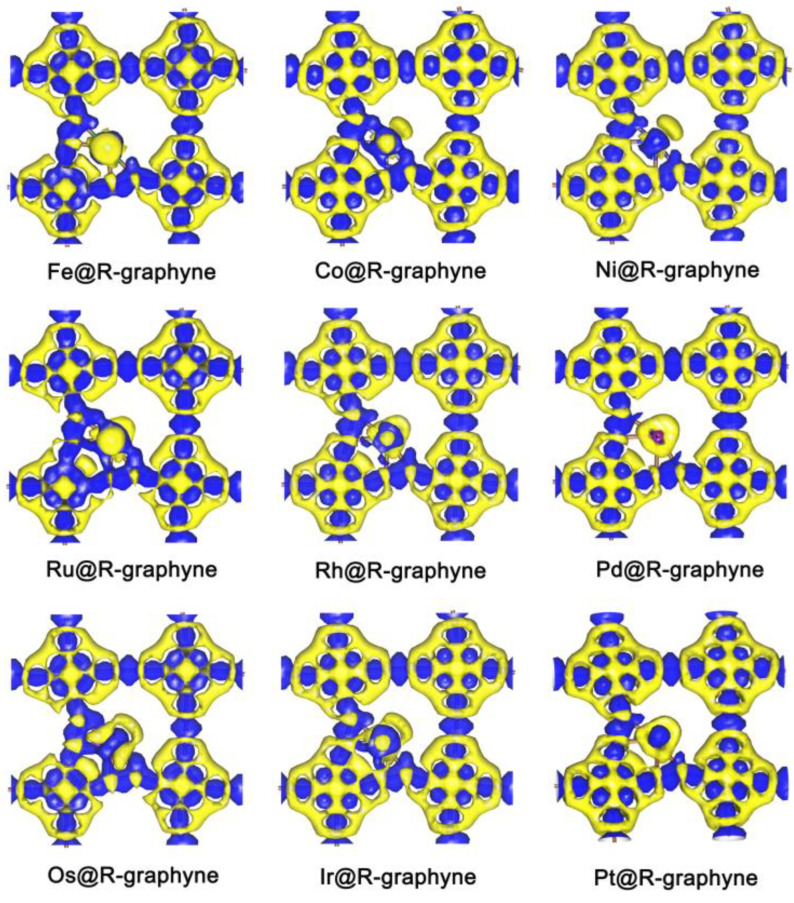
The electron density for TM@R-graphyne (TM = Fe, Co, Ni, Ru, Rh, Pd, Os, Ir and Pt) where the blue areas and yellow area mean the concentration of electron density and the depletion of electron density, respectively.

Obviously, embedding the TM atoms in Group VIIIB can be considered an effective strategy to improve the HER catalytic activity of 2D R-graphyne completely composed of anti-aromatic structural units. The relevant C atoms near the TM-doping sites can be used as highly active sites in HER. Generally, shorter distances between the TM and C atoms can achieve better HER activity at the T_C_ site. The enhancement of HER activity can be attributed to the fact that introducing the TM atoms of Group VIIIB can induce an electron transfer process between TM and its adjacent C atoms (Figure 4), which can effectively adjust the electron density on the relevant C atoms, thus endowing H* with an appropriate adsorption state. 

### 2.3. Evaluation of OER Catalytic Activity of TM@R-graphyne

**Figure 5:**
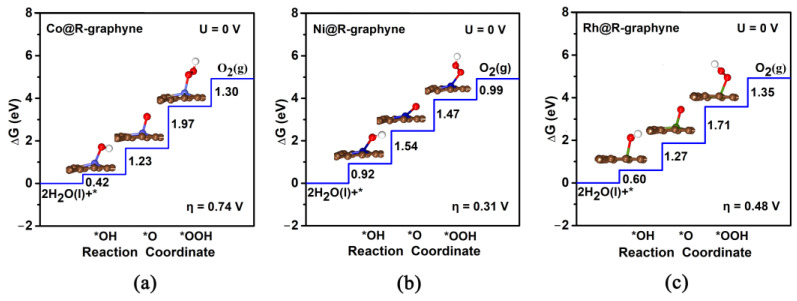
(**a**–**c**) The Gibbs free energy diagrams for OER on TM@R-graphyne (TM = Co, Ni and Rh) at an electrode potential of 0 V, where * denotes TM site.

In addition to HER activity, we investigate the OER electrocatalytic performance of R-graphyne doped with TM atoms of Group VIIIB by calculating the Gibbs free energy difference of four elementary steps (ΔG_1_, ΔG_2_, ΔG_3_ and ΔG_4_) to evaluate the overpotential of OER (η_OER_). It is noteworthy that the adsorption of intermediates (*O, *OH and *OOH) at the TM site on TM@R-graphyne (TM = Fe, Os, Pd and Pt) can lead to severe structural deformation, thus they are excluded. Finally, five possible candidates can be obtained, including Ru@R-graphyne, Co@R-graphyne, Rh@R-graphyne, Ir@R-graphyne and Ni@R-graphyne (Figure 5 and Figure S5).

For all four TM@R-graphyne systems (TM = Co, Ru, Rh and Ir), the third step (O*→*OOH) can be regarded as the potential-determining step (PDS) in the OER process (Figure 5a,c, and Figure S5). Among them, the calculated η_OER_ values of Ru@R-graphyne (1.43 V) and Ir@R-graphyne (1.16 V) can be largely positive, indicating the inert OER catalytic activity. Comparatively, Co@R-graphyne (0.74 V) can have certain OER activity, and especially Rh@R-graphyne can exhibit considerably high OER performance considering the very low η_OER_ value of 0.48 V. As for Ni@R-graphyne, the second step (OH*→*O) can be considered as the PDS step of OER (Figure 5b), and the calculated η_OER_ value can be as small as 0.31 V, indicating excellent OER catalytic activity. It is worth mentioning that the overpotentials of Ni@R-graphyne (0.31 V) and Rh@R-graphyne (0.48 V) can be comparable to or even lower than those of the state-of-the-art IrO_2_ (0.56 V) [12] and other previously reported MoC_2_ (0.45 V) [16] and RuO_2_ (0.42 V) [49] systems. Obviously, embedding Ni and Rh atoms in Group VIIIB can significantly improve the OER catalytic performance of R-graphyne.

**Figure 6:**
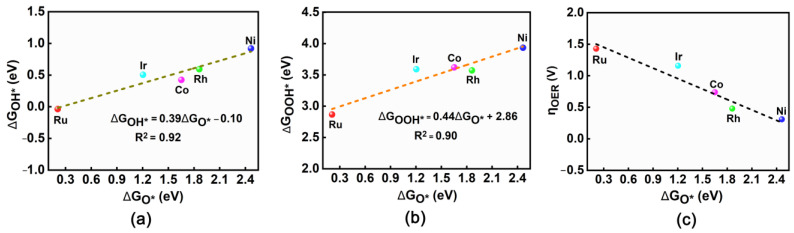
Linear relationship between ΔG_O*_ vs. G_OH*_ (**a**) and ΔG_O*_ vs. G_OOH*_(**b**) for TM@R-graphyne (TM = Co, Ni, Ru, Rh and Ir), (**c**) the relationship between η and ΔG_O*_ for TM@R-graphyne.

Generally, the relationship between the Gibbs free energies (ΔG_OH*_, ΔG_O*_ and ΔG_OOH*_) of three intermediates can be used to obtain the appropriate descriptors and thus reveal the OER trend of catalysts [50,51,52,53,54,55]. As shown in Figure 6a, ΔG_OH*_ can be specified as a function of ΔG_O*_, which can be expressed as ΔG_OH*_ = 0.39ΔG_O*_ − 0.10 eV. Clearly, ΔG_OH*_ can exhibit a strong linear correlation with ΔG_O*_ considering the high determination coefficient (R^2^) of 0.92. Moreover, ΔG_OOH*_ can also scale linearly with ΔG_O*_, which can be expressed as ΔG_OOH*_ = 0.44ΔG_O*_ + 2.86 eV with an R^2^ of 0.90 (Figure 6b). Obviously, ΔG_O*_ can be adopted as an effective descriptor for OER performance, because ΔG_OOH*_ and ΔG_OH*_ show strong linear correlation with ΔG_O*_ at the same time. 

**Figure 7:**
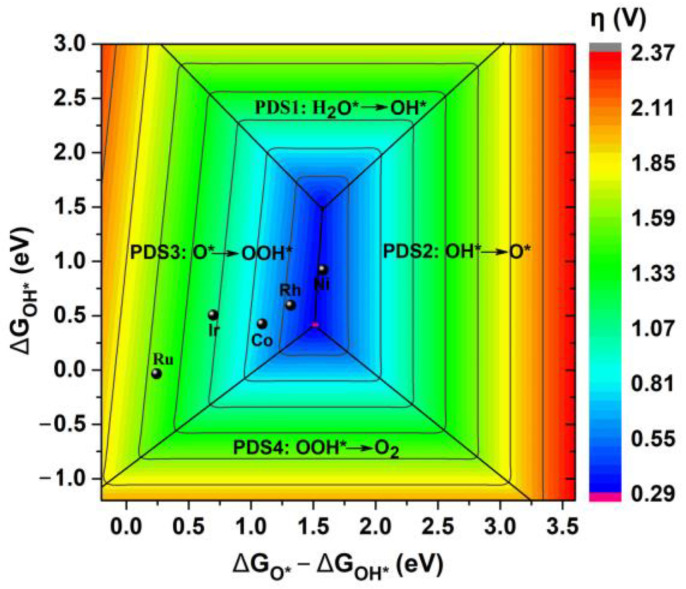
The 2D colored contour plot of OER activity for TM@R-graphyne (TM = Co, Ni, Ru, Rh and Ir) by showing the η values as a function of the Gibbs free energies.

Subsequently, we have drawn the diagram describing the relationship between η_OER_ and ΔG_O*_, as shown in Figure 6c. It can be found that along with the increase in ΔG_O*_ value, the calculated η_OER_ value can gradually decrease for these five TM@R-graphyne (TM = Ru, Co, Rh, Ir and Ni) systems, where doping Ni can bring the smallest η_OER_ value (0.31 V). Further, a contour map reflecting the trend of OER activity is also provided, which is divided into four regions based on the potential-determining step (Figure 7). The pink region in Figure 7 predicts the limiting overpotentials η of the TM@R-graphyne series, which can be as small as 0.29 V. It is worth mentioning that the newly designed Rh@R-graphyne (0.48 V) can be close to the limit value, and especially Ni@R-graphyne (0.31 V) can be very close to it, indicating considerably high OER catalytic performance. 

**Figure 8:**
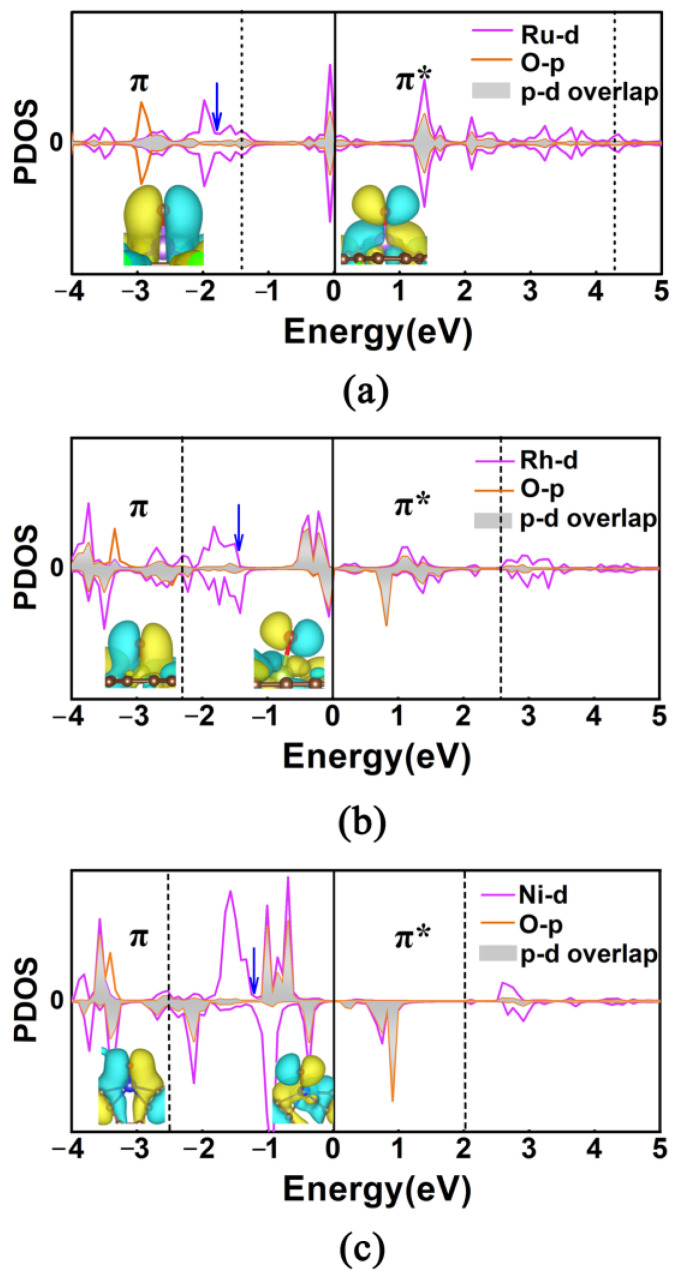
Partial density of states (PDOS) about the p orbitals of O atoms and d orbitals of TM atoms after the O* adsorption of Ru@R-graphyne (**a**), Rh@R-graphyne (**b**), and Ni@R-graphyne (**c**). The Fermi level energy is set as zero (black line). Inset: the molecular orbitals of the O atom adsorbed at the TM site in different energy ranges are marked by black dash line. Blue arrow represents the p-d overlap center.

From the above discussions, we can understand that the adsorption strength of O* plays a crucial role in determining the OER catalytic performance of TM@R-graphyne. In order to better understand that the doping of Ni and Rh atoms can bring the appropriate adsorption state of O*, we have conducted an in-depth bonding analysis on the intercation between O* and Ni/Rh center. For comparison, the TM@R-graphyne with poor catalytic activity in the OER is also considered by sampling Ru@R-graphyne. Figure 8 shows the partial density of states (PDOSs) of the p orbitals of O atoms and d orbitals of relevant TM atoms, where bond properties between O* and the metal site are presented by drawing different ranges of molecular orbitals. It can be found that when the doped Ru atom is used as an adsorption site, the center of overlapping O-p and Ru-d orbitals is in the π bonding region (Figure 8a), which can lead to a relatively strong interaction between O* and the TM site, thus resulting inthe poor OER activity. In contrast, when Rh atom is doped, the overlapping center of the O-p and Rh-d orbitals will move into the π* antibonding region (Figure 8b). The existence of an antibonding characteristic will effectively weaken the interaction strength between O* and the TM site, which can result in a relatively sutiable adsorption state of O*, thus significantly improving the catalytic activity of the OER. Further, when Ni is introduced into R-graphyne, the overlapping center of the O-p and Ni-d orbitals in the π* antibonding region can continue to move towards the Fermi level (Figure 8c), indicating that the antibonding characteristics can be increased to some extent, which will bring a more appropriate adsorption state of O*, thus further enhancing the OER activity.

In general, in addition to HER activity, the OER catalytic performance of 2D R-graphyne composed entirely of anti-aromatic structural units can also be effectively improved by doping the TM atoms in Group VIIIB. In particular, it is highly anticipated that both the Ni@R-graphyne and Rh@R-graphyne systems can be used as promising bifunctional electrocatalysts for HER and OER.

## 3. Computational Methods

The generalized gradient approximation (GGA) with the Perdew–Burke–Ernzerhof exchange correlation functional [56] is used to perform all the density functional theory (DFT) calculations of the studied systems within the framework of the Vienna ab initio simulation package (VASP) [57,58]. Spin-polarized calculations have been considered in this study. The cut-off energy for the plane-wave basis was set as 400 eV and a semi-empirical van der Waals (vdW) correction proposed by Grimme (DFT-D2) was employed to account for the dispersion interactions [56,59]. The ion-electron interaction was described by the projected enhancement wave (PAW) potential [60]. The 3 × 3 × 1 k-points were used for the structural optimization and 84 k-points were used to calculate the density of states (DOSs). The convergence criteria of total energy and force were 10^−4^ eV and 0.02 eV A^−1^, respectively. A vacuum space of 20 Å was applied along the z-direction to avoid the interactions between adjacent images. In addition, the nucleus-independent chemical shift (NICS) [61,62] calculations were conducted at the B3LYP/6-31G(d) level using the Gaussian 09 program package [63] to evaluate the aromatic properties of the relevant carbon rings in the studied system.

In order to evaluate the stability of transition-metal-doped R-graphyne system, the binding energy (E_b_) is calculated according to the following formula:

E_b_ = E_TM@R-graphyne_ − E_TM_ − E_R-graphyne_ (1)

where E_TM@R-graphyne_, E_TM_ and E_R-graphyne_ represent the total energy of TM@R-graphyne, single transition metal atom (TM) and the pristine R-graphyne, respectively. A more negative E_b_ value can mean higher stability.

The HER performance can be evaluated by calculating the free energy of the reaction (ΔG_H*_) according to the following formula:

∆G_H*_ = ∆E_H*_ + ∆ZPE − T∆S (2)

Here, ∆E_H*_ represents the energy difference of H* adsorption. ∆ZPE and ∆S are the changes of zero-point energy and entropy of H* adsorption, respectively. In this study, T∆S and ∆ZPE were obtained by following the scheme proposed by Nørskov et al. [46]. Specifically, ∆S was calculated by using the equation ∆S = S(H*) − 1/2S(H_2_) ≈−1/2S(H_2_), because the vibrational entropy of H* can be very small and negligible. Considering that TS (H_2_) is 0.410 eV for H_2_ at 298 K and 1 atm, the corresponding T∆S was determined as −0.205 eV. In addition, the equation ∆ZPE = ZPE(H*) − 1/2ZPE(H_2_) was applied to estimate ∆ZPE for H*. It is worth mentioning that our computed ZPE(H_2_) value is about 0.295 eV, which is close to the value reported by Nørskov et al. [46]. 

The OER process is usually carried out through the four-electron transfer steps, which can be summarized as follows:

H_2_O + *→*OH+ H^+^ + e^−^ (3)

*OH→*O + H^+^ + e^−^ (4)

H_2_O + *O→*OOH + H^+^ + e^−^ (5)

*OOH→* + O_2_ + H^+^ + e^−^ (6)

The Gibbs free energy change (ΔG) of each elemental step can be obtained by the following expression:

ΔG = ΔE + ∆ZPE − T∆S + ΔG_U_ + ΔG_pH_ (7)

where ∆E is the adsorption energy for the relevant intermediates involving OH, O and OOH. ΔZPE and ΔS are the zero-point energy change and the change in entropy, respectively. ΔG_U_ = −neU, where U is the electrode potential related to the standard hydrogen electrode. ∆G_pH_ = k_B_TIn10 × pH is the correction for Gibbs free energy depending on the concentration of H^+^ ions, and pH = 0 was used in this study. Moreover, the ΔG_i_ of each reaction step of OER can be expressed as ΔG_1_ = ΔG_OH*_, ΔG_2_ =ΔG_O*_ −ΔG_OH*_, ΔG_3_ =ΔG_OOH*_ −ΔG_O*_ and ΔG_4_ = 4.92 − ΔG_OOH*_. The overpotential for OER (η_OER_) was calculated according to Nørskov’s assumption [49]:

η_OER_ = max{ΔG_1_, ΔG_2_, ΔG_3_, ΔG_4_}/e − 1.23 (8)

where ΔG_i_ (i = 1–4) and 1.23 represent the Gibbs free energy change for step (i) and the equilibrium potential.

## 4. Conclusions

Through the detailed DFT calculations, the electrocatalytic HER and OER performance of R-graphyne and TM@R-graphyne (TM = Fe, Ru, Os, Co, Rh, Ir, Ni, Pd and Pt) has been systematically investigated. The following interesting findings can be mainly obtained:

(1) Compared with the inert graphene, 2D R-graphyne can exhibit certain HER electrocatalytic activity, which is mainly due to the anti-aromaticity of all its structural unit rings. Therefore, designing the 2D carbon allotrope completely composed of anti-aromatic structural units can be considered as an effective strategy to boost the HER catalytic activity of related carbon materials.

(2) Embedding the Group VIIIB atoms (i.e., Fe, Ru, Os, Co, Rh, Ir, Ni, Pd and Pt) can be considered as an effective approach to improve the HER catalytic activity of R-graphyne, where the C-atoms near the TM-doping site can usually be used as highly active sites. In particular, Fe@R-graphyne, Os@R-graphyne, Rh@R-graphyne and Ir@R-graphyne can exhibit higher HER activity in comparison with other TM@R-graphynes due to the formation of more active sites. Closer distances between the relevant C atoms and the TM-doping site can generally endow them with the better HER catalytic activity. The improvement of HER performance can be due to the electron transfer process between the TM and its adjacent C atoms, which effectively regulates the electron density on the relevant C atoms, thus bringing the appropriate adsorption state of H*.

(3) Doping Co, Ni and Rh atoms in Group VIIIB can effectively enhance the OER catalytic performance of 2D R-graphyne. In particular, Ni@R-graphyne and Rh@R-graphyne can be identified as promising electrocatalysts, being capable of driving OER under very small overpotentials of 0.31 V and 0.48 V, respectively. ΔG_O*_ can be considered to be a good descriptor for their OER activities. Further, we analyzed the reasons for the different OER activities of TM@R-graphyne systems from the perspective of molecular orbital theory. In addition, Ni@R-graphyne and Rh@R-graphyne are also expected to become bifunctional electrocatalysts for HER and OER.

Coupled with good conductivity and high structural stability, these 2D nanostructures based on R-graphyne can be considered as new and promising catalysts for HER or OER. All these intriguing findings can provide important insights for promoting the development of carbon-based electrocatalysts for water splitting. 

## Data Availability

Not applicable.

## Supplementary Materials

The following supporting information can be downloaded at https://www.mdpi.com/article/10.3390/molecules28093888/s1, Figure S1: Three different C-C bonds are labeled, as well as two possible adsorption sites (S1 and S2) of TM atom; Figure S2: The optimized configurations of TM@R-graphyne (top view and side view), in which the doped TM atoms are Fe (a), Co (b), Ni (c), Ru (d), Rh (e), Pd (f), Os (g), Ir (h) and Pt (i), respectively; Figure S3: The total energy curve of ab initio molecular dynamics (AIMD) simulation for TM@R-graphyne at 500 K, where the inset is the configuration after 5000 fs; Figure S4: The density of states (DOS) of TM@R-graphyne, where the Fermi energy was set to zero (black dash line); Figure S5: The Gibbs free energy diagrams for OER on Ru@R-graphyne and Ir@R-graphyne at an electrode potential of 0 V; Table S1: The optimized lattice constants (a = b) and the calculated binding energy (E_b_) for the TM@R-graphyne; Table S2: The calculated ΔG_H*_ (eV) values for H* at the different adsorption sites (S_ad_) on TM@R-graphyne; The coordinate files of the intermediate structures are in Figure 5.

## Sample Availability

Samples are available from the authors.
