# Supplementary material for: Theoretical Investigation of HER and OER Electrocatalysts Based on the 2D R-graphyne Completely Composed of Anti-Aromatic Carbon Rings
Source: Molecules. 2023 May 5;28(9):3888. doi: 10.3390/molecules28093888 (PMC10180217; doi:10.3390/molecules28093888)
Supplement: Supplementary file 1 [file molecules-28-03888-s001.zip › molecules-2373112-supplementary.pdf]

Supplementary Information

Theoretical Investigation of HER and OER Electrocatalysts Based on the 2D R-graphyne Completely Composed of Anti-Aromatic Carbon Rings

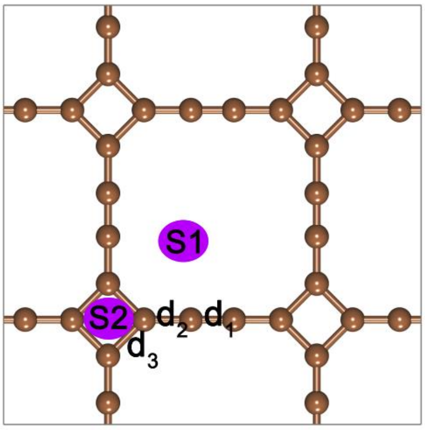

Figure S1. Three different C-C bonds are labeled, as well as two possible adsorption sites (S1 and S2) of TM atom.

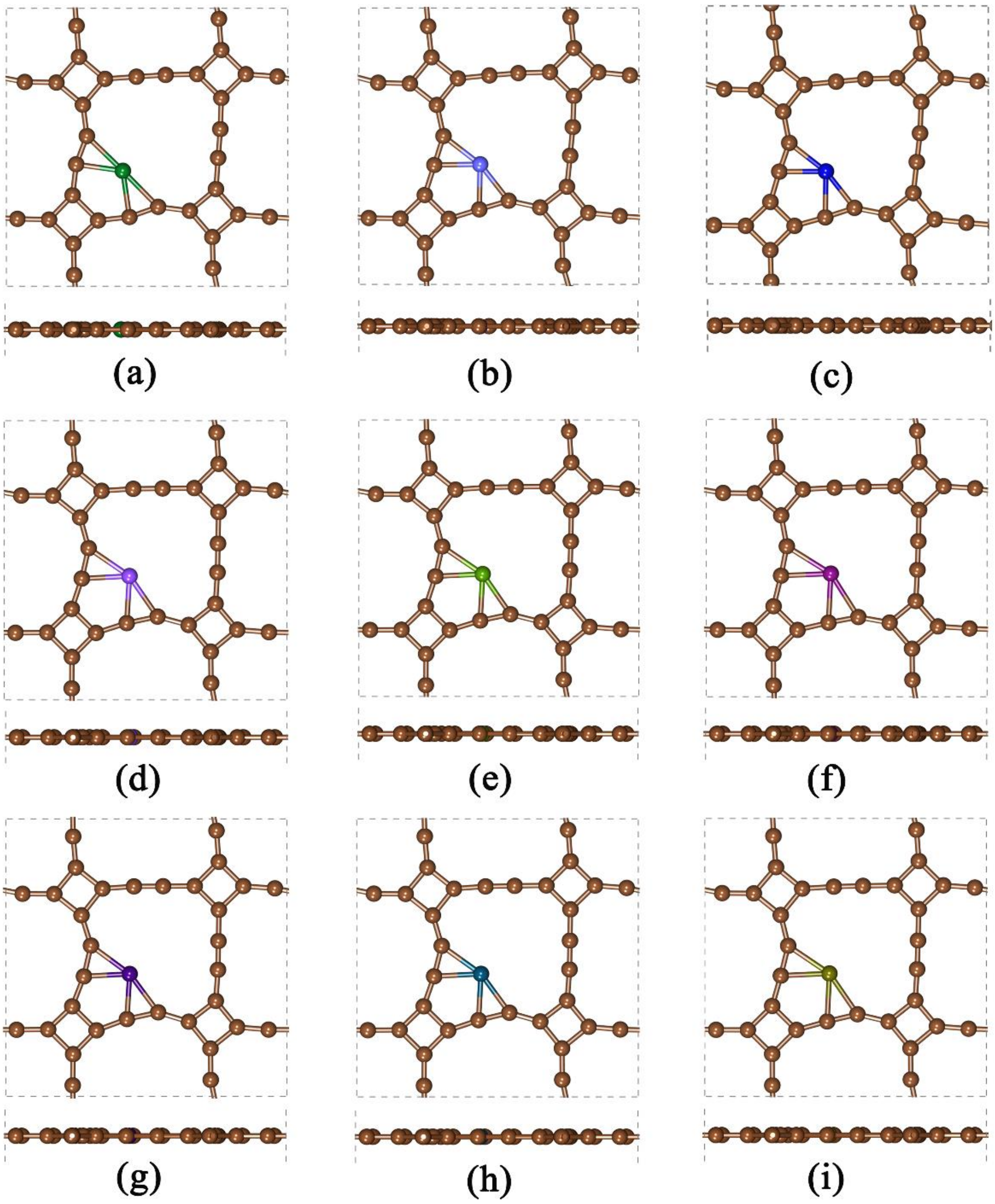

Figure S2. The optimized configurations of TM@R-graphyne (top view and side view), in which the doped TM atoms are Fe (a), Co (b), Ni (c), Ru (d), Rh (e), Pd (f), Os (g), Ir (h) and Pt (i), respectively.

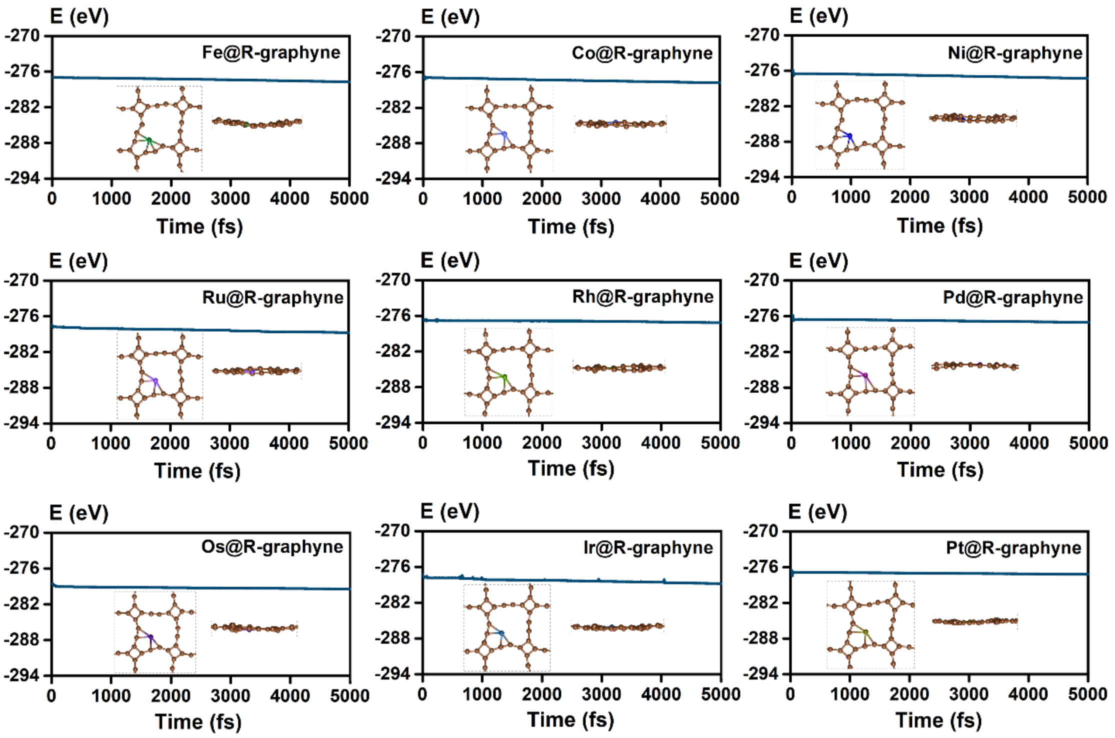

Figure S3. The total energy curves of ab initio molecular dynamics (AIMD) simulation for TM@R-graphyne at 500 K, where the insets are the configurations after 5000fs.

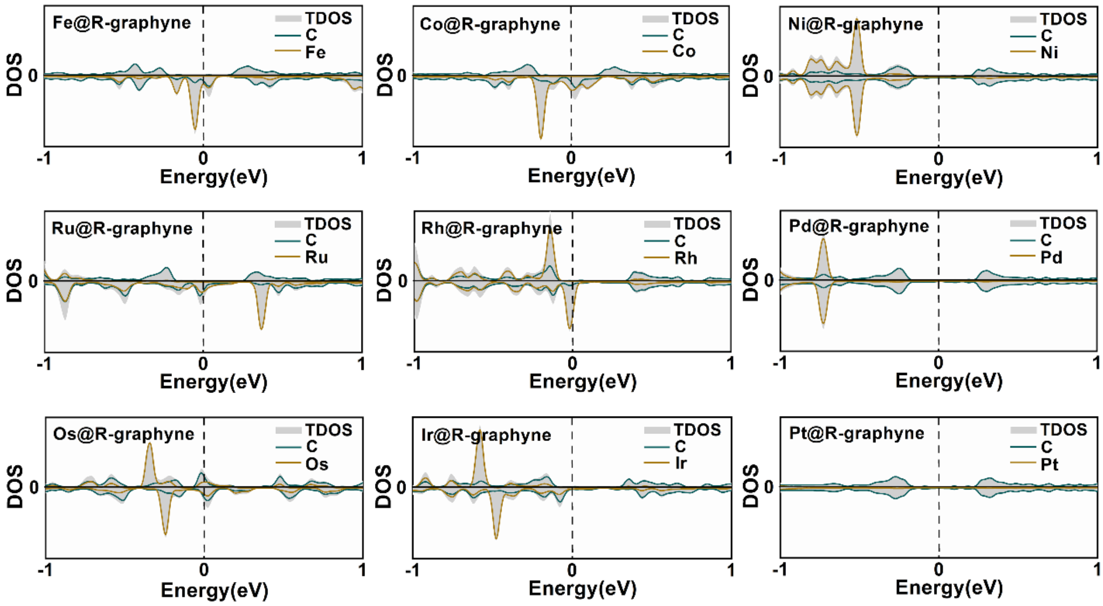

Figure S4. The density of states (DOSs) of TM@R-graphyne, where the Fermi energy was set to zero (black dash line).

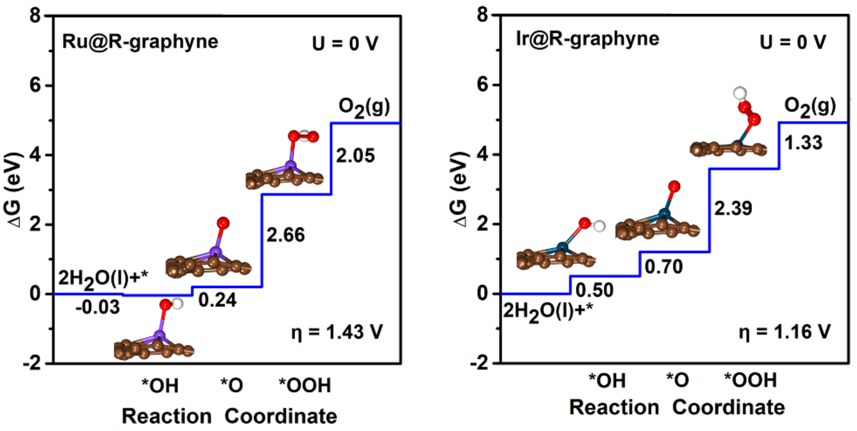

Figure S5. The Gibbs free energy diagrams for OER on Ru@R-graphyne and Ir@R-graphyne at an electrode potential of 0 V.

Table S1. The optimized lattice constants (a=b) and the calculated binding energy (E_b) for the TM@R-graphyne.

System	a = b (Å)	E_b (eV)
Fe@R-graphyne	a=b=11.987	-2.509
Co@R-graphyne	a=b=11.951	-3.077
Ni@R-graphyne	a=b=11.934	-3.400
Ru@R-graphyne	a=b=11.993	-4.321
Rh@R-graphyne	a=b=11.990	-3.600
Pd@R-graphyne	a=b=11.994	-2.631
Os@R-graphyne	a=b=11.990	-4.561
Ir@R-graphyne	a=b=11.980	-4.773
Pt@R-graphyne	a=b=11.981	-4.190

Table S2. The calculated ΔG_{H^*} (eV) values for H* at the different adsorption sites (S_{ad}) on TM@R-graphyne.

S_{ad}	ΔG_{H^*} (eV)								
	TM=Fe	TM=Co	TM=Ni	TM=Ru	TM=Rh	TM=Pd	TM=Os	TM=Ir	TM=Pt
T_{TM}	0.142	0.069	0.811	-0.501	-0.497	0.887	-0.778	-0.581	0.586
T_{C1}	-1.246	-0.715	-0.600	-1.106	-0.920	0.240	-0.140	-0.005	0.267
T_{C2}	0.239	0.442	0.448	-0.104	-0.118	0.351	0.272	0.246	0.361
T_{C3}	-0.140	0.068	0.210	-0.014	-0.037	0.282	-0.263	-0.099	0.303
T_{C4}	0.122	0.369	0.379	0.316	0.283	0.415	0.196	0.241	0.429
T_{C5}	0.181	0.393	0.408	0.328	0.285	0.368	0.334	0.294	0.387
T_{C6}	0.235	0.437	0.400	0.425	0.399	0.392	0.461	0.398	0.403
T_{C7}	0.250	0.449	0.405	0.493	0.457	0.421	0.487	0.449	0.424
T_{C8}	0.237	0.414	0.372	0.503	0.416	0.412	0.496	0.404	0.415

The coordinate files of the intermediate structures in Figure 5

Co@R-graphyne-OH
C Co O H
1.00000000000000
11.9250001907000005 0.0000000000000000 0.0000000000000000
0.0000000000000000 11.9259996414000007 0.0000000000000000
0.0000000000000000 0.0000000000000000 20.0000000000000000
C Co O H
32 1 1 1
Direct
0.2316012316863859 0.1449320714431060 0.4931596432449747
0.2330285969139873 0.3178498496176594 0.4966987562556332
0.3172236452010229 0.2320399281476177 0.4972715055114728
0.1443347578026389 0.2335340360351049 0.4925375512830753
0.4264268148569820 0.2638928540528121 0.5047637807252853
0.0316725604014367 0.2380176254753793 0.4930829727468753
0.2664848379522730 0.4266607357330518 0.5039714791899216
0.2348409402216388 0.0321073458755784 0.4941815655881651
0.7222446700453815 0.1825380878270471 0.4988449212430157
0.7376948268174346 0.3550357528997496 0.5001643691404769
0.8161640476325817 0.2616977223196899 0.4975930956427780
0.6434111083281627 0.2779418053406334 0.5006040655532922
0.9282102993081807 0.2503194647581723 0.4952569922979025
0.5299842319505461 0.2958519901012522 0.5030889784737855
0.7489962076241837 0.4673554744597074 0.5026673205518891
0.7269924458137352 0.0694727462715317 0.4995230722296662
0.2777800292903439 0.6439811677292352 0.5016120831552152
0.2598642375975643 0.8166860941165847 0.4987026825456712
0.3539270394230010 0.7393173925611073 0.5015734490945952
0.1815853272839625 0.7222068410363077 0.4993616719369818
0.4662158036372303 0.7515121096405355 0.5038063697848044
0.0684726993311458 0.7265525829086408 0.4993746707457925
0.2474865505605209 0.9287702438196682 0.4963541965701236
0.2967157293603570 0.5309343147901276 0.5033801411224923
0.7677632736615158 0.6831485660394181 0.5042029144656990
0.7681019887526258 0.8564703000091491 0.5020758271619427
0.8555462976214814 0.7684000267571268 0.5018818715462978
0.6821274718343826 0.7693679501989164 0.5044715161407237
0.9679991272408504 0.7558909421393664 0.5001381151731410
0.5692969381412391 0.7648376790205806 0.5048731078083267
0.7562511433113126 0.9690216602583006 0.5004865526653963
0.7625646162237834 0.5703644058537932 0.5040442907750851
0.4325331812459858 0.4319169143271347 0.5255468393451346
0.4861719436381072 0.4853170037617869 0.6032002036848464
0.5450953972880230 0.5416065016741377 0.5966335385995224

Co@R-graphyne-O
C Co O
1.00000000000000
11.9300003052000001 0.0000000000000000 0.0000000000000000
0.0000000000000000 11.9280004501000008 0.0000000000000000
0.0000000000000000 0.0000000000000000 20.0000000000000000

C Co O
32 1 1
Direct
0.2344854200432382 0.1475819884496122 0.4971964466277364
0.2348910725769666 0.3205960767125504 0.5011997643187517
0.3194834322301482 0.2359039020260005 0.5009013430053227
0.1465157236105735 0.2356263194856744 0.4974894055855984
0.4289738094303125 0.2693680414594648 0.5046819951234240
0.0342941294078531 0.2387922152527398 0.4964864620930282
0.2687950742401174 0.4299472985986905 0.5052312661518137
0.2376730345726154 0.0353129811875548 0.4960786666540246
0.7238590676180440 0.1831017399516239 0.4982324887432852
0.7413082610812730 0.3556444405149902 0.4989794453596429
0.8188261017295964 0.2613480831881523 0.4975267365304212
0.6465005769615305 0.2792004014161093 0.4996515738589738
0.9304909746256761 0.2495544114916349 0.4966128822816152
0.5333685969962808 0.2998334768436192 0.5019297318219650
0.7534568271641328 0.4677755323070779 0.4997173126133660
0.7280374667972926 0.0699730274271339 0.4985463808105067
0.2790751040733482 0.6474527121848107 0.5001806285693784
0.2610032681373184 0.8198452363873941 0.4976490648082401
0.3553816414396263 0.7423717783817562 0.4992382207099392
0.1829291197816681 0.7247028311347540 0.4986354370245255
0.4675091921802190 0.7544163490883593 0.4998654531257157
0.0698159060591302 0.7286076103062932 0.4988615062522659
0.2489397670890464 0.9315375311249315 0.4964181589553701
0.2996990137922823 0.5342665773598796 0.5025540933957439
0.7694486192453736 0.6836555192736891 0.5004373069296559
0.7693836155361720 0.8570053419586944 0.4997373427778764
0.8567697177041573 0.7695120768525873 0.4997980419524331
0.6834198309067560 0.7697542409300658 0.5003956061591611
0.9693490603224969 0.7576362259125211 0.4991776038155700
0.5707783867710291 0.7660385849953553 0.5001841240072592
0.7574644186356311 0.9695966017696176 0.4989795308264081
0.7655021832810763 0.5710004515959393 0.5001619219922938
0.4335109531168211 0.4337974825472865 0.5190589212177221
0.4678606998422174 0.4672629978834512 0.5952551179009604

Co@R-graphyne-OOH
C Co O H
1.00000000000000
11.9300003052000001 0.0000000000000000 0.0000000000000000
0.0000000000000000 11.9270000457999998 0.0000000000000000
0.0000000000000000 0.0000000000000000 20.0000000000000000
C Co O H
32 1 2 1
Direct
0.2341610563930558 0.1457055178089505 0.4894794050940445
0.2320535970494806 0.3188243882705999 0.4942702139099852
0.3185778795134724 0.2344670917309872 0.4929754303545512
0.1455200952552555 0.2325595110633615 0.4906497406884470
0.4271094567980929 0.2667337007197016 0.5019757381738268

0.0326416474622430	0.2350127712785664	0.4928248563487357
0.2618400724663363	0.4278637185676467	0.5037698167401318
0.2384385451794816	0.0329984651462222	0.4910366307497864
0.7230359125900616	0.1803345493468950	0.4995618207019717
0.7398356555768441	0.3526935241012942	0.5026583053484167
0.8171731669247352	0.2588415340029523	0.4988838498279234
0.6444573162087512	0.2763855716558677	0.5028053865737968
0.9293373002837362	0.2469206404393280	0.4957432323201251
0.5317483931184593	0.2958975558023532	0.5048071671603481
0.7516689768080380	0.4649807941055896	0.5045615836570214
0.7273451453906096	0.0673417186446667	0.4991025829970798
0.2774296041555087	0.6447122649426642	0.5027962544045409
0.2607143864985573	0.8174853731986208	0.4982661970553126
0.3541190093718112	0.7393054161722593	0.5015522092875592
0.1818203849602318	0.7233944716177141	0.5002858410866093
0.4664704299584379	0.7506609634068596	0.5029583648698067
0.0688815928742558	0.7275326798904064	0.5005806270582610
0.2494716304441934	0.9294968014540497	0.4943622623087957
0.2954537711502375	0.5312147241924595	0.5057526164314248
0.7683417445526587	0.6808999841339972	0.5046199063595869
0.7677843048052826	0.8542537807325510	0.5013668509559271
0.8556285730772692	0.7667420477201317	0.5020333150784736
0.6823761755415856	0.7667189936842393	0.5039508459929303
0.9680916057296862	0.7556542035894068	0.5009041498072877
0.5695732078583701	0.7624322223768626	0.5038269174847433
0.7560733300845685	0.9666925378814825	0.4995502370927370
0.7639981004198242	0.5681382835507990	0.5052950295490274
0.4287890050529995	0.4306751913849355	0.5261609278384876
0.4598119834681882	0.4532036424611295	0.6129557784831150
0.4981312012724004	0.5665417154061619	0.6296307401522022
0.5158056547052727	0.5553536115183023	0.6770651770569612

Ni@R-graphyne-OH
C Ni O H
1.00000000000000

11.9399995804000003	0.0000000000000000	0.0000000000000000
0.0000000000000000	11.9359998702999999	0.0000000000000000
0.0000000000000000	0.0000000000000000	20.0000000000000000

C	Ni	O	H
32	1	1	1

Direct

0.2305813276378451	0.1455469239188386	0.4930133251750481
0.2316143039993003	0.3184153231045547	0.4948592323797764
0.3162949810417846	0.2334255351041837	0.4949653869612795
0.1433254030041632	0.2335872885024510	0.4928264387147014
0.4254933102202907	0.2650428302456039	0.5015991190705139
0.0306252852731617	0.2374181660251594	0.4946714459112669
0.2627093271350302	0.4280081257454317	0.5016466623649346
0.2337493180759981	0.0329653156450365	0.4949854237946765
0.7216124382870837	0.1816519471306606	0.5020269264226206
0.7375941370731013	0.3540602533522299	0.5040473150245512
0.8153259105391050	0.2604931739636379	0.5010772599101729

0.6427885452496805	0.2770298278904393	0.5044759634489595
0.9273586063010003	0.2491252494709604	0.4977583824285129
0.5295657662153271	0.2951995808484765	0.5053486972398511
0.7497305899292083	0.4663159096862782	0.5049849922215706
0.7262075163266597	0.0685632086083857	0.5015441014007167
0.2746087957983798	0.6453631804786765	0.5047979680521768
0.2584193412857737	0.8178426857706400	0.5013867889090969
0.3518514613206822	0.7398004757715889	0.5043300331109509
0.1793304308574569	0.7241391565728952	0.5023033664406028
0.4640966807946896	0.7521080187515828	0.5047914969213331
0.0663753852884147	0.7282586185769445	0.5018006457673327
0.2466148727374975	0.9297425108737525	0.4980776611665028
0.2900127886701800	0.5320215557926661	0.5052023963421154
0.7654104864416343	0.6822246499657361	0.5046320771972406
0.7658672076300163	0.8553503369829427	0.5025457445783750
0.8531059281332216	0.7676499216289562	0.5027308471812612
0.6797661242597181	0.7685148508022375	0.5044317254286902
0.9655567514539560	0.7560149761800434	0.5017781729325417
0.5669781794354241	0.7652127235553191	0.5047383288030765
0.7547209419251049	0.9679895692330571	0.5015865736335359
0.7615319901991597	0.5694917615450480	0.5052181919355547
0.4311916774388307	0.4311843720577747	0.5162835508743334
0.5031703400313284	0.5118829569023750	0.5806846687046000
0.5762238829898005	0.4805089653154455	0.5922191465515380

Ni@R-graphyne-O
C Ni O
1.00000000000000

11.9399995804000003	0.0000000000000000	0.0000000000000000
0.0000000000000000	11.9289999007999992	0.0000000000000000
0.0000000000000000	0.0000000000000000	20.0000000000000000

C Ni O
32 1 1
Direct

0.2314008478705672	0.1455327963451669	0.4934657818985055
0.2311267205937522	0.3183842846460078	0.4962040802407598
0.3159605598488739	0.2346462950863159	0.4958922149727268
0.1432041243527597	0.2327533413091229	0.4938200032110420
0.4254744742272871	0.2665175609164548	0.5013365706057277
0.0306551155458861	0.2360603063043651	0.4953338358789358
0.2613750518475103	0.4282612797957600	0.5017211599456620
0.2359496436831625	0.0329452907608645	0.4945137332074957
0.7217091235044391	0.1813900452597992	0.5008403647990602
0.7377185717999168	0.3541179896283237	0.5032414515652525
0.8154917424092805	0.2603744400012172	0.5004639197604805
0.6431852672382639	0.2769889256251113	0.5031507324365441
0.9273842857310894	0.2485550977218345	0.4978653250265962
0.5297096641733748	0.2927557783554658	0.5036323154696406
0.7505726912304076	0.4662858495869003	0.5044973259873966
0.7264115979624056	0.0682779401061101	0.5001882786710165
0.2768587102978252	0.6453114729908277	0.5028539984107820
0.2611728845805015	0.8176981754723465	0.4995687713131341

0.3545228631085067	0.7392823367121664	0.5022363143916730
0.1818102054094071	0.7241058922193604	0.5008041658156189
0.4666860703994722	0.7513025695964530	0.5033438687797638
0.0687944610147647	0.7285351130872257	0.5008372800684766
0.2495544772051361	0.9296541528913687	0.4968075638475292
0.2908936906812483	0.5313604097876233	0.5036410490971049
0.7684345147379268	0.6819683323986909	0.5044025643290060
0.7678791346428773	0.8553242127247479	0.5018664418830343
0.8555436514730074	0.7679175670155128	0.5022193362992140
0.6823190528080353	0.7678398703077877	0.5039830109970771
0.9681325663263856	0.7566528323915528	0.5011521540525514
0.5696357101613475	0.7642828137234678	0.5039028727275718
0.7558491353959137	0.9678844480820955	0.5003942948251444
0.7647431799822505	0.5691736039027702	0.5048810834486545
0.4379343873042178	0.4385962839191568	0.5168118092801992
0.5215558654521861	0.5254926653280122	0.5615562567566215

Ni@R-graphyne-OOH
C Ni O H
1.00000000000000

11.9320001601999994	0.0000000000000000	0.0000000000000000
0.0000000000000000	11.9289999007999992	0.0000000000000000
0.0000000000000000	0.0000000000000000	20.0000000000000000

C	Ni	O	H
32	1	2	1

Direct

0.2325450413969450	0.1435054935513746	0.4919893708486300
0.2341706302378977	0.3166622641748157	0.4941709048249070
0.3184892983262344	0.2310436787592110	0.4937664444344799
0.1455458910064047	0.2318311033340053	0.4922109346975617
0.4279689470352938	0.2609447465859937	0.5007572182222325
0.0329171288667251	0.2359200239900144	0.4945945828092134
0.2661160440706332	0.4257158464629068	0.5021671935213093
0.2356874140728048	0.0307982260428300	0.4945938747042944
0.7242340828579908	0.1798644138375871	0.5017048047129387
0.7391272287843523	0.3525343460562253	0.5038196402055020
0.8175843141128274	0.2594235139290237	0.5010731821106092
0.6449674450731278	0.2748744547915586	0.5039649657299869
0.9296468263910118	0.2482987301098835	0.4978368948232588
0.5315428236417963	0.2904654136181933	0.5043987007483837
0.7510739776020706	0.4648098039089003	0.5044258481066749
0.7288399089680644	0.0669289280675306	0.5008629743986630
0.2773945519808743	0.6430166634346228	0.5069866368663642
0.2606689930854793	0.8156662519364833	0.5023501977552111
0.3542431856438398	0.7379013822030135	0.5056643313090878
0.1817621763280716	0.7216841038653223	0.5038412536100575
0.4664977333240859	0.7500527972760536	0.5050752176714624
0.0688881553830371	0.7262514397726353	0.5027182491091817
0.2486879278790895	0.9275870434671692	0.4982329573593344
0.2947190227921546	0.5298321632046803	0.5076669914585925
0.7683864313414818	0.6805197596154310	0.5039708497683754
0.7681754273291879	0.8536926880139565	0.5017778797379360

0.8558093382497017	0.7662845295822022	0.5021800096965203
0.6823421474712954	0.7663416507415682	0.5036728099480631
0.9682566501108640	0.7549703335402251	0.5019380790185421
0.5695743597195573	0.7623230559666673	0.5042598048034268
0.7569886398472990	0.9661805753295237	0.5008258278068281
0.7644844243184428	0.5677624296328158	0.5044722481971161
0.4313159144563375	0.4296439848801220	0.5189383824065592
0.5152582260457462	0.4826994113860115	0.5885831800984029
0.4719427897366468	0.5799937338781950	0.6225035831793481
0.4850468315126350	0.5584850880532335	0.6692540543009456

Rh@R-graphyne-OH
C Rh O H
1.00000000000000

11.9899997711000008	0.0000000000000000	0.0000000000000000
0.0000000000000000	11.9899997711000008	0.0000000000000000
0.0000000000000000	0.0000000000000000	20.0000000000000000

C Rh O H
32 1 1 1
Direct

0.2398353315018473	0.1535624110816876	0.5017664877097943
0.2385616875051266	0.3259318142278945	0.5033918280528648
0.3249343715313338	0.2409591258625848	0.5035073020466201
0.1524402606034977	0.2399248241865355	0.5016842353663779
0.4341175101877453	0.2727336638663836	0.5030772657465010
0.0399636847642460	0.2397786567573159	0.5003677294966280
0.2675099495431635	0.4359726333060860	0.5027419188809944
0.2406985244061540	0.0413375606369899	0.5003591949842109
0.7303701840993321	0.1746696843022478	0.5007664810639758
0.7476696123926951	0.3465004198878858	0.5011144435217693
0.8243741401045702	0.2529714420378544	0.5005258249468374
0.6524186118403361	0.2706261442261683	0.5014479447186253
0.9365515358465069	0.2436045720106151	0.5000863408991156
0.5417906627125706	0.2963535490197651	0.5015590527682525
0.7559145243240522	0.4587210827712593	0.5009928975666903
0.7309361086060910	0.0620397914715303	0.5003246610376391
0.2668786568252914	0.6539716226637192	0.4996936400811965
0.2525595270154041	0.8258872952315482	0.4993268538444904
0.3445831722234894	0.7473490660861050	0.4996349646307092
0.1725385420417158	0.7333035726000262	0.4992794696857326
0.4569090741413888	0.7542356312910932	0.4998652628349591
0.0600352738498667	0.7352191002969182	0.4993970575425458
0.2447345852692694	0.9379262804764490	0.4996390645282075
0.2893753240396097	0.5428087260555952	0.5002999669534027
0.7590463511165073	0.6746632334737277	0.5005258930169511
0.7578055316577322	0.8473892285962473	0.5000829894477007
0.8454150344703314	0.7610857283768881	0.5001080004938137
0.6727713246395000	0.7597000513999396	0.5003519918010276
0.9577104966474255	0.7539288231550700	0.4997170326792187
0.5603040695292760	0.7575295420649013	0.5000771570592605
0.7494439817677591	0.9596611518094873	0.5000252879874100
0.7584013432644225	0.5621941681736027	0.5008306707906338

0.4410358809517451 0.4432237649669713 0.5037522480416434
0.4710538421763166 0.4790420368734693 0.5961157545821405
0.5237613664036835 0.4234536837554392 0.6131330951920625

Rh@R-graphyne-O
C Rh O
1.00000000000000
11.9899997711000008 0.0000000000000000 0.0000000000000000
0.0000000000000000 11.9899997711000008 0.0000000000000000
0.0000000000000000 0.0000000000000000 20.0000000000000000
C Rh O
32 1 1
Direct
0.2379211488015935 0.1512406294867074 0.4989409009544571
0.2385248288096639 0.3235188784890572 0.5027869349361102
0.3229978292288172 0.2383923634710315 0.5027592289864726
0.1508635404310756 0.2391775590933732 0.4989215533736454
0.4333230045490315 0.2683714431365302 0.5046959814637006
0.0388448364156152 0.2405061640852873 0.4971919854850907
0.2695256626554149 0.4336474818529168 0.5051486806494684
0.2384888959682094 0.0392926791609803 0.4973303729298838
0.7304746048574136 0.1738614295113082 0.4977558927358477
0.7447786072364037 0.3461000349108129 0.4983453373769237
0.8234612425167397 0.2535006458633132 0.4972863945846146
0.6516614196446963 0.2683118428760130 0.4988148508077166
0.9352453332673367 0.2451648162304929 0.4967879131263986
0.5397695627955665 0.2888766194528495 0.5009429591491092
0.7523312846969245 0.4582191294345263 0.4989506098341875
0.7323611121348393 0.0611501460017359 0.4982349138728853
0.2686836737275998 0.6520877536030013 0.4996150681585020
0.2524695159002283 0.8239169807526636 0.4977872097432362
0.3456026007460133 0.7458770406331960 0.4993347202328801
0.1735979828679313 0.7301999338072338 0.4980357590947239
0.4576817948472595 0.7540547438137253 0.5000759156248911
0.0608228657133766 0.7315447726945422 0.4982318733855631
0.2433907066052662 0.9356275204381318 0.4971769742639027
0.2909523568151018 0.5403794330441140 0.5018812064117087
0.7590607360763059 0.6739651018019202 0.4997856380485085
0.7596156701595250 0.8465759606207290 0.4993230497914378
0.8462416265889562 0.7594235561922560 0.4991654255224640
0.6734901118217756 0.7601071937106879 0.5000497529544605
0.9586687254673164 0.7510486711175292 0.4986007325460880
0.5611201647208469 0.7587184763645597 0.5002229685916587
0.7518757095080169 0.9590192012628508 0.4987682326199651
0.7569760488306845 0.5616417805804456 0.4994341098061372
0.4417779421515925 0.4424717611046615 0.5155977358771241
0.4800789054428597 0.4738383354008205 0.5993191070602399

Rh@R-graphyne-OOH
C Rh O H
1.00000000000000

```
     11.9849996566999994         0.0000000000000000         0.0000000000000000
      0.0000000000000000        11.9899997711000008         0.0000000000000000
      0.0000000000000000         0.0000000000000000        20.0000000000000000
   C      Rh     O      H
    32     1     2     1
Direct
  0.2410683380686179  0.1503741249480652  0.5057066686942482
  0.2419183400955086  0.3227200152349908  0.5084855522897657
  0.3271448964550939  0.2366066168665930  0.5087073232211043
  0.1549299264692528  0.2374092947309670  0.5054399510615077
  0.4369516304768139  0.2659285785128201  0.5069528305922090
  0.0423549808888943  0.2383259987685227  0.5020144343453929
  0.2727190435316629  0.4323566595221190  0.5067858810510089
  0.2413811953149495  0.0378044077898934  0.5028971279172170
  0.7344588929061627  0.1713329634919818  0.4992154246522145
  0.7485572975425132  0.3432920781563820  0.4985960566653909
  0.8267254876589422  0.2514823296180076  0.4986597559814600
  0.6548003990691202  0.2657488917788450  0.4995834202216222
  0.9391912875734193  0.2433800989931369  0.4998354966687386
  0.5437642030646013  0.2875806948680457  0.5017692866670957
  0.7558435812928320  0.4558522041702353  0.4988580306598653
  0.7363063342066755  0.0589814005686890  0.4998914285500951
  0.2707578285188066  0.6502617825338805  0.5004340186171250
  0.2550366312083794  0.8222168298237322  0.5003311828626416
  0.3473838575102311  0.7446333417450679  0.4999269215906298
  0.1755362493881286  0.7291821493218956  0.5004900896348642
  0.4599183307728250  0.7525172659126582  0.4998551831493823
  0.0631757252485484  0.7304707265973247  0.5008336781388940
  0.2460778998649622  0.9345670996642830  0.5013248207547047
  0.2943783295908275  0.5395355087712250  0.5020018517258449
  0.7610100433228756  0.6718188487207897  0.5003978575999413
  0.7618443914635646  0.8443567143085949  0.5007189346552643
  0.8486025568649391  0.7570606277511135  0.5007574999873602
  0.6759740175202663  0.7574497547243214  0.5005197318377375
  0.9606750691767717  0.7490103622553017  0.5008397119465064
  0.5631752502080890  0.7560652645606621  0.5000703725621490
  0.7546326817324990  0.9565317054146807  0.5003743762930890
  0.7592871205444106  0.5590973340275373  0.4995296340187865
  0.4449137931504447  0.4396278546739238  0.5065807106678522
  0.5030625844695051  0.4722513341736574  0.5939453337604497
  0.4544392896225340  0.5693346513735191  0.6245954460819407
  0.4039225712063333  0.5353745346265512  0.6575639688758903
```
